# Detection of micro-pinhole defects on surface of metallized ceramic ring combining improved DETR network with morphological operations

**DOI:** 10.1371/journal.pone.0321849

**Published:** 2025-04-22

**Authors:** Yisong Xiao, Xian Wang, Yunlong Liu, Tianlong Yang, Jigang Wu

**Affiliations:** 1 School of Mechanical Engineering, Hunan University of Science and Technology, Xiangtan, China; 2 State Grid Hunan Electric Power Company Research Institute, Changsha, China; IIIT Kurnool: Indian Institute of Information Technology Design and Manufacturing Kurnool, INDIA

## Abstract

Metallized Ceramic Ring is a novel electronic apparatus widely applied in communication, new energy, aerospace and other fields. Due to its complicated technique, there would be inevitably various defects on its surface; among which, the tiny pinhole defects with complex texture are the most difficult to detect, and there is no reliable method of automatic detection. This Paper proposes a method of detecting micro-pinhole defects on surface of metallized ceramic ring combining Improved Detection Transformer (DETR) Network with morphological operations, utilizing two modules, namely, deep learning-based and morphology-based pinhole defect detection to detect the pinholes, and finally combining the detection results of such two modules, so as to obtain a more accurate result. In order to improve the detection performance of DETR Network in aforesaid module of deep learning, EfficientNet-B2 is used to improve ResNet-50 of standard DETR network, the parameter-free attention mechanism (SimAM) 3-D weight attention mechanism is used to improve Sequeeze-and-Excitation (SE) attention mechanism in EfficientNet-B2 network, and linear combination loss function of Smooth L1 and Complete Intersection over Union (CIoU) is used to improve regressive loss function of training network. The experiment indicates that the recall and the precision of the proposed method are 83.5% and 86.0% respectively, much better than current mainstream methods of micro defect detection, meeting requirements of detection at industrial site.

## 1. Introduction

The metallized ceramic ring is a kind of novel electronic apparatus coated on surface or deposited on metal layer of ceramic materials, widely applied in communication, power and other fields [1–3]. The pinhole defect is a common surface defect on the metal layer of metallized ceramic ring. It has a very small area, where the background texture is complex, so it is extremely difficult to detect the pinhole defect. Manual inspection is currently the main method for detecting tiny pinhole defects on the surface of metallized ceramic rings. However, it is also difficult for manual inspection to detect defects with tiny area such as pinhole defects, with FPR and FNR remaining high [4–7]. It is necessary to develop a high-accuracy, stable and reliable automatic detection technology for pinhole defects of metallized ceramic rings.

In recent years, defect detection technology based on machine vision has developed fast and is widely used in many fields. Machine vision defect detection can be divided into two categories: traditional methods and deep learning-based methods [8,9]. For the traditional method, the features of the graph are extracted firstly, and the defective areas in the image are identified according to a certain criterion; traditional methods can be divided into three categories: statistical method, spectral method and model-based method. The statistical method counts and evaluates the pixels of the defective image, and detects surface defects by analyzing the spatial distribution of pixels. Prasitmeeboon et al. [10] introduced a novel method for detecting defects in particleboard. This method utilizes traditional machine learning techniques to analyze the bivariate color histogram of the particleboard for rapid determination of the presence of defects. The spectral method transforms the defective image in the spatial domain, frequency domain and other domains, and realizes the surface defect detection based on the different responses of the normal area and the defective area. In order to improve the detection accuracy of low-contrast line defects, Xie et al. [11] proposed a display line defect detection method based on color feature fusion. Saliency maps are generated using color features, and morphological operations are applied to segment defect regions from the background. The model-based method achieves defect detection by establishing a mathematical model of the objected surface defect, mapping the original texture distribution of the defective image to a low-dimensional space, and detecting anomaly by analyzing the deviation of the mapping result from normal surface model. Traditional method of machine learning often falls into this category. Zhou et al. [12] proposed a wavelet transform multiscale filtering (WTMF) defect detection strategy based on wavelet transform and multiscale filtering algorithms to detect defects on the bottom of glass bottles.

Currently, although traditional methods still have advantages in certain circumstances, deep learning-based methods have far outperformed traditional methods in most applications. Deep learning-based method is the one most frequently studied in the field of defect detection [13,14]. Compared with traditional methods, the defect detection method based on deep learning introduces deep neural network to autonomously learn defects of deep-level abstract features. Ayon et al. [15] Proposed a metal surface flaw detection method using LMViT, achieving superior accuracy over CNNs and other transformer models, especially for subtle defects.Luo et al. [16] proposed an aluminum surface defect detection method based on deep learning, which improved the defect detection accuracy by using Inception V4 as the backbone feature extraction network. On the basis of YOLOv4, Zhuxi et al. [17] proposed a more lightweight method for detecting surface defects of aluminum strip. Based on MobileNet, they redesigned the backbone network of YOLOv4, and added channel attention and spatial attention mechanisms.

The existing algorithms have remarkable effects on those defects with a large proportion of image or large size, but their performance of detecting small defects is not satisfactory. The metallized ceramic ring is small, and the defective area is smaller; the pixel resolution of picture is 2448×2048, while the pixel of pinhole defect is only about 8×8, offering little information of feature that can be used for defect detection. In addition, the scale of the metallized ceramic ring dataset is small, making it difficult to fully train the deep learning model and fully exert the advantages of the deep learning method.

In order to improve the performance of micro defect detection, researchers have made a lot of attempts. Some researchers improved the structure of convolutional neural network to enhance the granularity of feature extraction capabilities, so that it can better capture the subtle features of micro defects. In order to detect small defects on the surface of chemical special steels, Wang et al. [18] proposed an improved defect detection algorithm based on YOLOv8. They introduced a ParC2Net (Parallel-C2f) structure for feature extraction, which accurately captures the subtle features of steel defects. Data augmentation has also proven to be an effective means of improving the performance of micro defect detection. By generating pseudo-defect datasets similar to real defects, it is possible to partially settle the problem of difficulty in model training due to insufficient data. Mohammeda et al. [19] proposed a conditional generative adversarial network (cGAN) for fabric defect data augmentation. This technique helps overcome the limitations of small or unvaried datasets, leading to improved defect detection accuracy and generalizability. In addition, the integration of deep learning and traditional image processing methods has also become an important research field of improving the detection performance of micro defects. Su et al. [20] proposed SRResNetYOLO, combining visual saliency, feature fusion, and ResNet-21 to detect small, multi-scale defects on metal gear end-faces using light reflection and image processing techniques. All the above methods can improve the detection accuracy of micro defect detection tasks to a certain extent, serving as a reference for this study.

In 2020, Carion et al. proposed a DETR network model based on Transformer architecture. Different from the traditional CNN-based detection model, DETR effectively models the global dependencies between object parts in the image through the self-attention mechanism, which makes it can better deal with complex image background and small object detection problems. Teng et al. [21] proposed a semi-supervised DETR model supplemented with convolutional augmentation for railway ballast bed defect detection, termed as Semi-Conv-DETR model. Dang et al. [22] devised a novel sewer defect detection system motivated by the state-of-the-art detection transformer (DETR) architecture, which views object localization as a set prediction topic.

In order to meet the requirements of pinhole defect detection on the surface of metallized ceramic rings, this Paper proposes a pinhole defect detection method on the surface of metallized ceramic rings combining improved DETR network with morphological algorithm. Firstly, the standard DETR network is improved to enhance the performance of detecting micro defects; this method also combines respective advantages of the deep learning network and traditional methods, and can be applied to inspection at industrial field.

The structure of this paper is as follows: Section 2 provides a detailed introduction to the material acquisition and the proposed method. In Section 3, we experiment and evaluate the performance of the proposed method and analyze the experimental results. The conclusion of the paper is discussed in Section 4.

## 2. Materials and methods

### 2.1 Data acquisition

**Fig 1.**
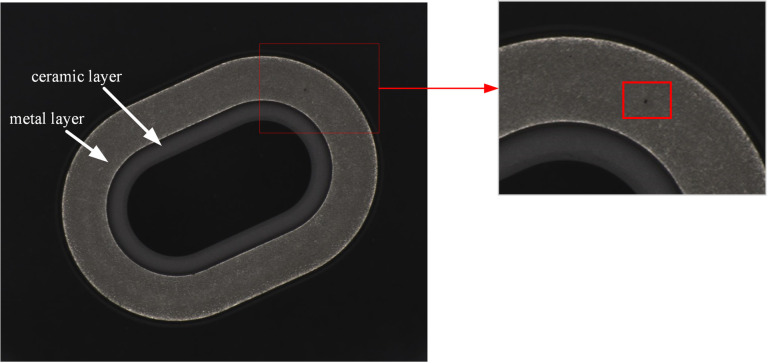
The metallized ceramic ring and the pinhole defects on the surface of its metal layer.

The metallized ceramic ring detected in this study, as shown in (Fig 1), consists of a metal layer and a ceramic layer, with length of 17.5 mm, width of 12.5 mm, and height of 3.5 mm. Pinhole defects only occur on the metal layer. A pinhole defect has an area of about 0.0001 square millimeters, only accounting for 0.002% of the surface area of the metal layer, and there is little feature information which can be used for defect detection. In addition, there are complex granular textures of different sizes and shapes on the surface of the metal layer, making it harder to identity pinhole defect.

The image acquisition of defects of metallized ceramic ring has been carried out at a plant of industrial ceramics located in middle of Hunan Province, China. The image acquisition device consists of a data acquisition table, an industrial camera, an LED ringlike light source and a lens, and the specific model and parameters are shown in Table 1. A total of 2000 frames were collected at the site, and each sample contained one or more pinhole defects, with a total of 2563 defects. The resolution of the sample image is 2448×2048 pixels, and the pixel area of the metallized ceramic ring is about 928800 px^2^, while the pixel area of the pinhole defect is only 64 px^2^, accounting for only 0.007% of the pixel area of the metallized ceramic ring.

**Table 1.** Model and parameters of each device in the acquisition device.

Name	Model	Performance parameters
Industrial camera	MER2-501-23GC	Pixel 2448(H)×2048(V)
Prime lens	HN-P-2528-6M-C2/3	Focal length 25mm
Ring light	HK-R100-40–22	Color temperature 6000K, Power 126W

### 2.2 Proposed method

**Fig 2:**
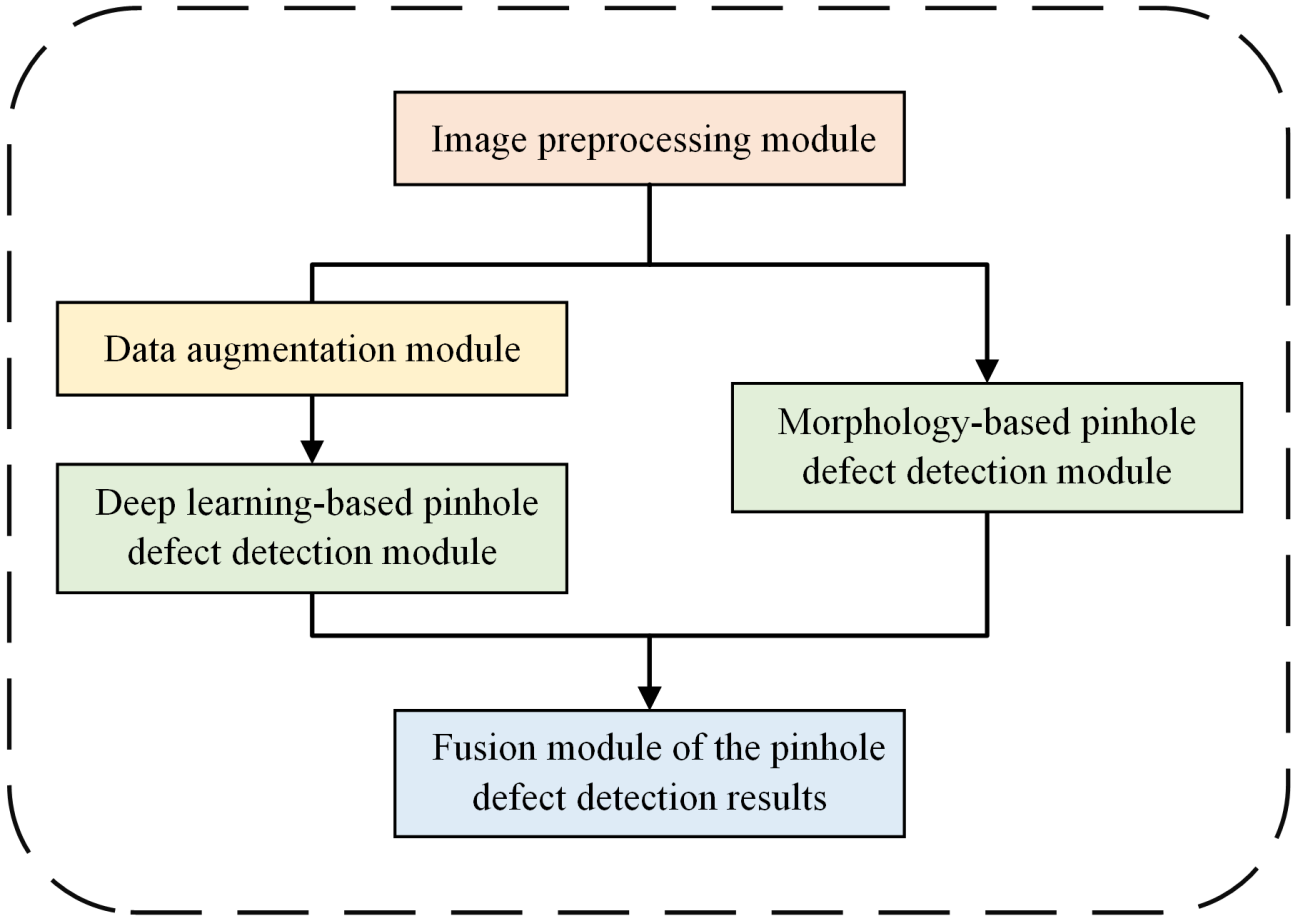
Schematic diagram of the pinhole defect detection module of the metallized ceramic ring.

With respect to the problems existing in the visual detection of micro pinhole defects on the surface of metallized ceramic ring, this paper proposes a method for detecting micro pinhole defects on the surface of metallized ceramic rings with improved DETR network and morphological algorithm. As shown in (Fig 2), the method can be divided into five modules: image preprocessing, data augmentation, morphology-based pinhole defect detection, deep learning-based pinhole defect detection, and combined detection results of pinhole defects. Firstly, the image preprocessing module is used to remove the ceramic layer and inhibit the interference of lighting conditions on the detection results. Then, the preprocessed images are input into the data augmentation module and the morphology-based pinhole defect detection module separately. In the data augmentation module, the quality of the dataset is improved by sample category balancing and data enrichment. The morphology-based pinhole defect detection module detects pinhole defects by way of connected component analysis (CCA). The sample image enriched by the data augmentation module will be input into the deep learning-based pinhole defect detection module, which uses an improved DETR network to detect pinhole defects. Finally, the detection results obtained independently by the two pinhole defect detection modules are respectively input into the fusion module of the pinhole defect detection results to combine the detection results verified by the aforesaid two detection modules, so as to obtain more accurate pinhole defect detection results.

#### 2.2.1 Image preprocessing and image segmentation.

**Fig 3:**
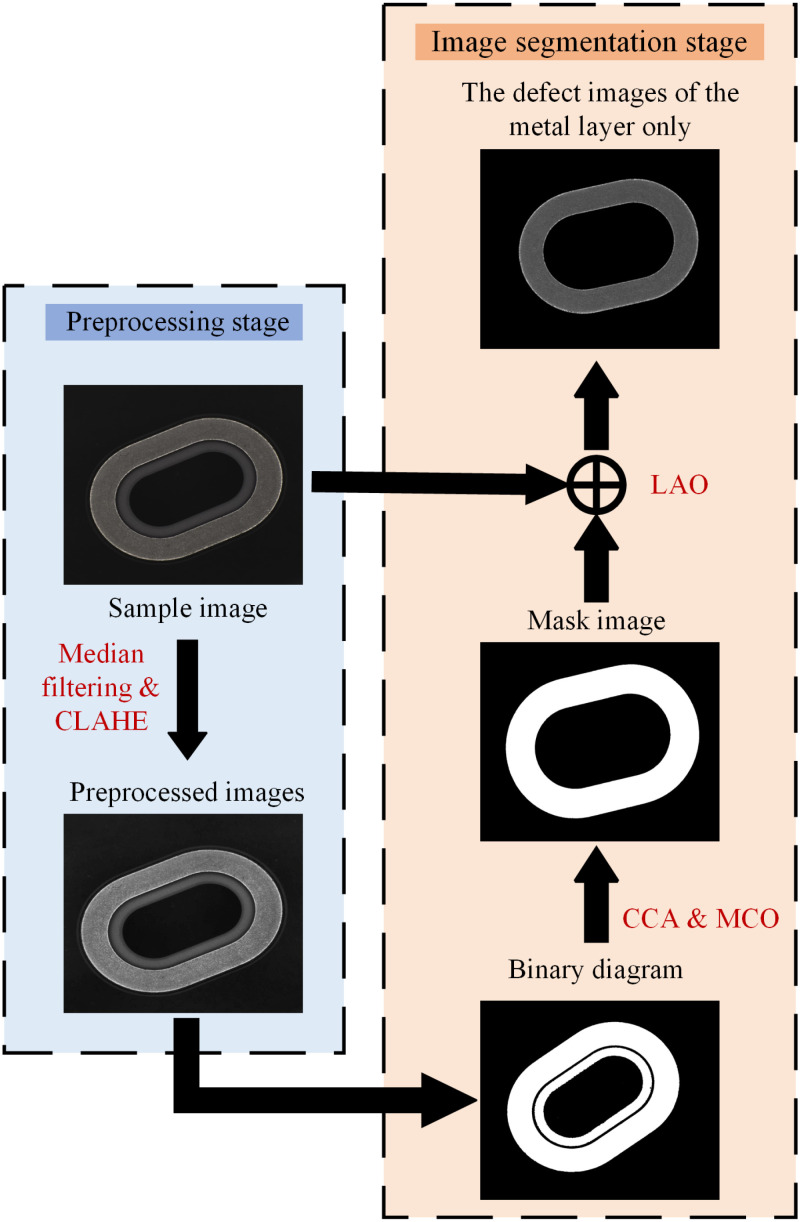
Sample image preprocessing and image segmentation process.

The images of the metallized ceramic ring sample collected at the site contain the ceramic layer and the metal layer, and the pinhole defects only occur on the metal layer. The ceramic layer without pinhole defects also has complex texture; in addition, both the metal and ceramic layers are sensitive to lighting conditions when acquiring defect images. In order to inhibit the illumination conditions and ceramic layers from interfering with the detection results, it is necessary to preprocess and segment the collected sample images; such process is shown in (Fig 3). During the preprocessing stage, the Contrast-Limiting Adaptive Histogram Equalization (CLAHE) method is used to enhance the contrast between the metal layer and the ceramic layer in the sample image, and median filtering is then used to suppress the noise caused by illumination conditions during the acquisition of defect images of the metallized ceramic ring.

During the image segmentation stage, the threshold segmentation is carried out on the preprocessed images by Otsu’s method (gray value of metal layer is 1, and 0 for other regions). Then, the mask image is obtained by the CCA and morphological closed operation (MCO), and then the mask image and the sample image are processed using the logical AND operation (LAO) to obtain the defect images of the metal layer only, which are then input into the morphology-based pinhole defect detection module and data enhancement module.

#### 2.2.2 Data augmentation.

When using deep learning model to detect defects, a high-quality training dataset can significantly influence the model performance. According to Subsection 2.1, although a lot of resources have been used to collect defect samples, only 2000 metallized ceramic ring samples have been collected at the site, with only 2563 pinhole defects; such dataset is much smaller than the large-scale public dataset with detection object as a general object. Therefore, in order to guarantee the effectiveness of the deep learning model, the quality of the dataset is improved by data augmentation, with a view to solve the issue of small sample size in the defect dataset of metallized ceramic ring.

**Fig 4:**
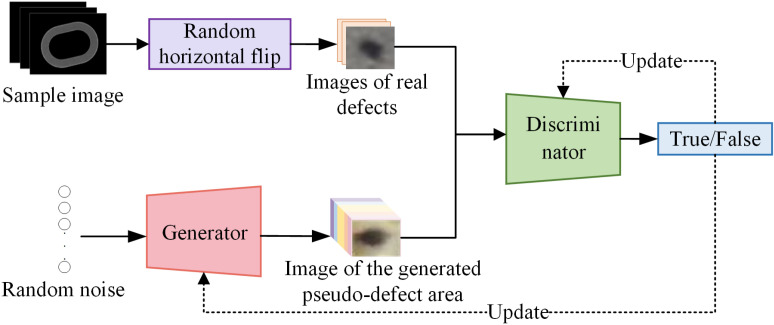
Basic framework of data augmentation.

By extracting and combining features of the original dataset, the Generative Adversarial Networks (GAN) generates pseudo-samples which are similar with the distribution of the original samples to the maximum extent, providing more samples for the object detection network [19,23]. The data augmentation diagram is shown in (Fig 4). Random horizontal flipping is used to increase the diversity and quantity of original images; then, GAN is used to generate pseudo-pinhole regions. Finally, the generated pseudo-pinhole region and the real pinhole defect area for training will serve as the training dataset of the subsequent object detection network. The numbers of pinhole defects before and after data augmentation are shown in Table 2.

**Table 2:** Numbers of pinhole defects before and after data augmentation.

Condition	Number of pinhole defects
Before data augmentation	2563
After data augmentation	3687

#### 2.2.3 Deep learning-based pinhole defect detection module.

The pinhole defect detection module based on deep learning aims at detecting pinhole defects using improved DETR network. The deep learning method has better adaptability when dealing with defect images with complex backgrounds and noise. The surfaces of the metal layers of some metallized ceramic rings contain many pinhole defects, and the surface texture of the metal layer will affect the choice of threshold during morphological detection, so for this kind of defect image, the deep learning method has better effect of detection.

**Fig 5:**
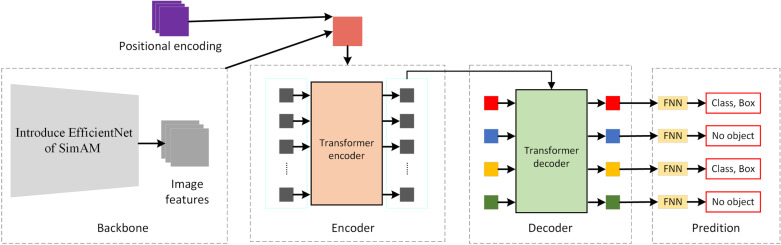
Improved DETR Network Model.

The deep learning-based pinhole defect detection module relies on an efficient and accurate object detection network. The DERT network is an end-to-end object detection method based on the Transformer architecture [24]. DETR network allows the model to focus on multiple locations simultaneously across the entire image by right of use global attention mechanism, helping to solve the problem of overlapping objects and indefinite number of objects. Although the DETR network performs outstanding in object detection on large-scale public datasets, the performance needs to be improved when the object is small. As can be seen from Subsection 1.1, the area of the pinhole defect is only 8×8 pixels, accounting for only 0.01% of the sample image. Therefore, in this Paper, the DETR network is improved after considering tiny pinhole defect on the surface of metallized ceramic rings, and the improved DETR network is shown in (Fig 5).

In order to improve DETR’s capacity of extracting micro defects on surfaces of metallized ceramic ring, the improved EfficientNet-B2 network in this Paper is substituted for backbone feature network ResNet-50 in original DETR network. As a kind of light model, EfficientNet optimizes the design of three dimensions, namely network depth, channel number and resolution, through compound scaling, reducing model parameters and computing amount while improving precision [25]. EfficientNet-B2 model structure is composed of 16 Mobile inverted bottleneck (MBConv) modules through piling, and SE attention mechanism idea has been added into each MBConv module; SE attention mechanism module adopts 1×1 convolution to adjust the feature image, and transfers high-dimension feature image into low-dimension feature vector through compression, so as to obtain global information among channels. In the vision, the channel region and space region co-exist, jointly promoting the selection of visual information; while SE attention module only pays attention to feature information among channels, without considering the special position information, which is critical for detection task of pinhole defects on surface of metallized ceramic ring, affecting the feature extraction to some extent. In order to strengthen the capacity of network feature extraction and improve network detection capacity, 3-D weight attention module SimAM is introduced to further optimize the EfficientNet-B2.

**Fig 6:**
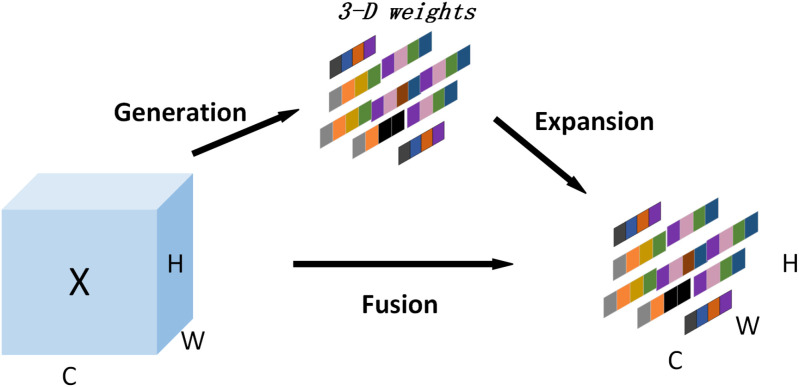
Principles of SimAM attention mechanism.

Different with CBAM, Coordinate and other mechanisms of mixed attention (considering channel and spatial dimensions), SimAM can directly estimate 3-D attention weight of feature image according to neuroscience theory, avoiding manual adjustment of network structure [26]. Refer to (Fig 6) for the theoretic map of SimAM attention mechanism.

**Fig 7:**
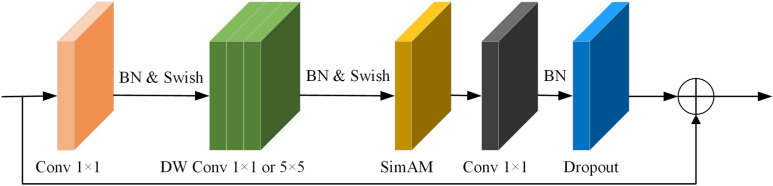
MBconv structure with SimAM.

SimAM has both channel and spatial dimensions, so SE attention module in MBconv module of backbone feature extraction network EfficientNet-B2 is substituted with SimAM attention module; refer to (Fig 7) for improved MBconv structure. Improved network pays attention to information of each channel, and reserves effective features in relation to defects on surface of metallized ceramic ring to the maximum extent.

The loss function in the network training process is an important factor influencing deep learning algorithm performance. Different with traditional CNN which adopts Anchor and NMS network, DETR network directly utilizes global modeling capacity of Transformer to obtain fixed prediction set, and gets best one-versus-one matching between real value and predicted value by calculating the loss between real value *y* and prediction set $\hat{y}$ and applying Hungarian algorithm $\left(L_{Hungarian}\right)$, as showed in Formula (1)


$$L_{Hungarian}\left(y,\hat{y}\right)=\sum_{i=1}^{N}\left[L_{cls}+L_{box}\left(b_{i},{\hat{b}}_{\hat{\sigma }\left(i\right)}\right)\right]$$ (1)

Of which, *N* represents predicted number of the object in the image; $L_{cls}$ represents classification loss of prediction box, calculating classification loss by applying cross-entropy function; $L_{box}$ represents the regression loss of the prediction box; $b_{i}$ represents the true bounding box of the object, including the four parameters: coordinates of the center point $\left(x,y\right)$, height $\left(h\right)$ and width $\left(w\right)$. ${\hat{b}}_{\hat{\sigma }\left(i\right)}$ represents the bounding box predicted by the model, including the four parameters: the coordinates of the center point $\left(x_{1},y_{1}\right)$ and height $\left(h_{1}\right)$ and width $\left(w_{1}\right)$.

The loss function of the standard DETR network is composed of the prediction box classification loss and the prediction box regression loss; the regression loss function adopts the linear combination of L1 Loss function and Generalized Intersection over Union (GIoU) function, but such two functions have problems such as unstable solution and insufficient accuracy of positioning small objects in the training process. The Smooth L1 loss function combines the advantages of the L1 and L2 loss functions. In tasks such as object detection and regression, it can usually provide better performance and a more stable training process. By considering parameters such as distance from center point and aspect ratio, the CIOU loss function can more accurately locate the position and shape of small objects, so as to improve the accuracy of the model’s detection of small objects. In order to further improve the accuracy of the model in the prediction box regression and improve the problems of instability in the model’s solution and insufficient accuracy in positioning small objects, the linear combination of Smooth L1 [27] function and CIoU [28] function() is used to replace the prediction box regression loss in the standard DETR model loss function:


$$L_{box}^{\prime }\left(b_{i},{\hat{b}}_{{\hat{\sigma }}_{\left(i\right)}}\right)=\lambda_{1}L_{1}\left(b_{i},{\hat{b}}_{{\hat{\sigma }}_{\left(i\right)}}\right)+\lambda_{2}L_{2}\left(b_{i},{\hat{b}}_{{\hat{\sigma }}_{\left(i\right)}}\right)$$ (2)

Of which, $\lambda_{1}�\lambda_{2}\in \mathbb{R}$ are hyperparameters; $L_{1}$ is Smooth L1 loss function; $L_{2}$ is CIoU loss function.

**Fig 8:**
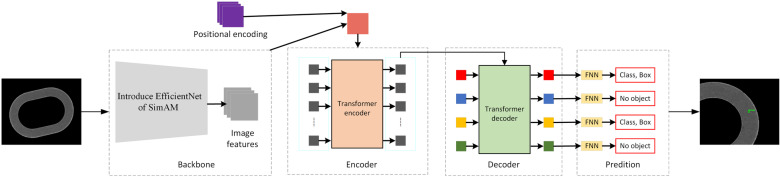
Pinhole defect detection procedure based on deep learning.

To make model focus on pinhole defect region and reduce interference from background, the defect images of metallized ceramic ring involved in metal layer only obtained from Subsection 1.2.1 are input into improved DETR network for detection; refer to (Fig 8) for the detection procedure.

#### 2.2.4 Morphology-based pinhole defect detection module.

**Fig 9:**
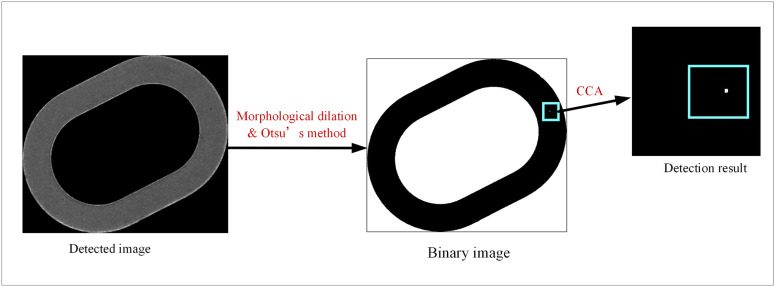
The morphology-based pinhole defect detection module flow.

The working process of the morphology-based pinhole defect detection module is shown in (Fig 9). In the defect image involving in only the metal layer obtained in Subsection 2.2.1, the main body of the metal layer accounts for a small proportion of the whole image, and when the Otsu’s method is directly applied to the defect image, too much black background area will affect the selection of the binarization threshold. For this purpose, it is necessary to acquire the region of interest (ROI) before the morphological method can be used to detect pinhole defects. By algorithm, the metal layer area is positioned in the defect image containing only the metal layer and is set as the ROI. Finally, the ROI region is extracted to obtain the detection image of this module.

The morphology-based pinhole defect detection module identifies tiny pinhole defects in the inspection image through CCA. Firstly, the Otsu method is used to binarize the detection image, so that the pinhole defect area exists in the form of a small area of white connected component. Then, the connected component with an area larger than the set threshold T is removed by the connectivity component analysis, and the remaining connected component with smaller area is the area where the pinhole defect is located. Finally, the morphological dilation of the detection image is carried out by using a rectangular structural element with the anchor position located at the geometric center and a size of 9×9 pixels (the center point of the structural element is the geometric center of the rectangle), so as to expand the area of the aforesaid connected component and ensure that the pinhole defects could be completely covered.

#### 2.2.5 Fusion module of pinhole defect detection result.

**Fig 10:**
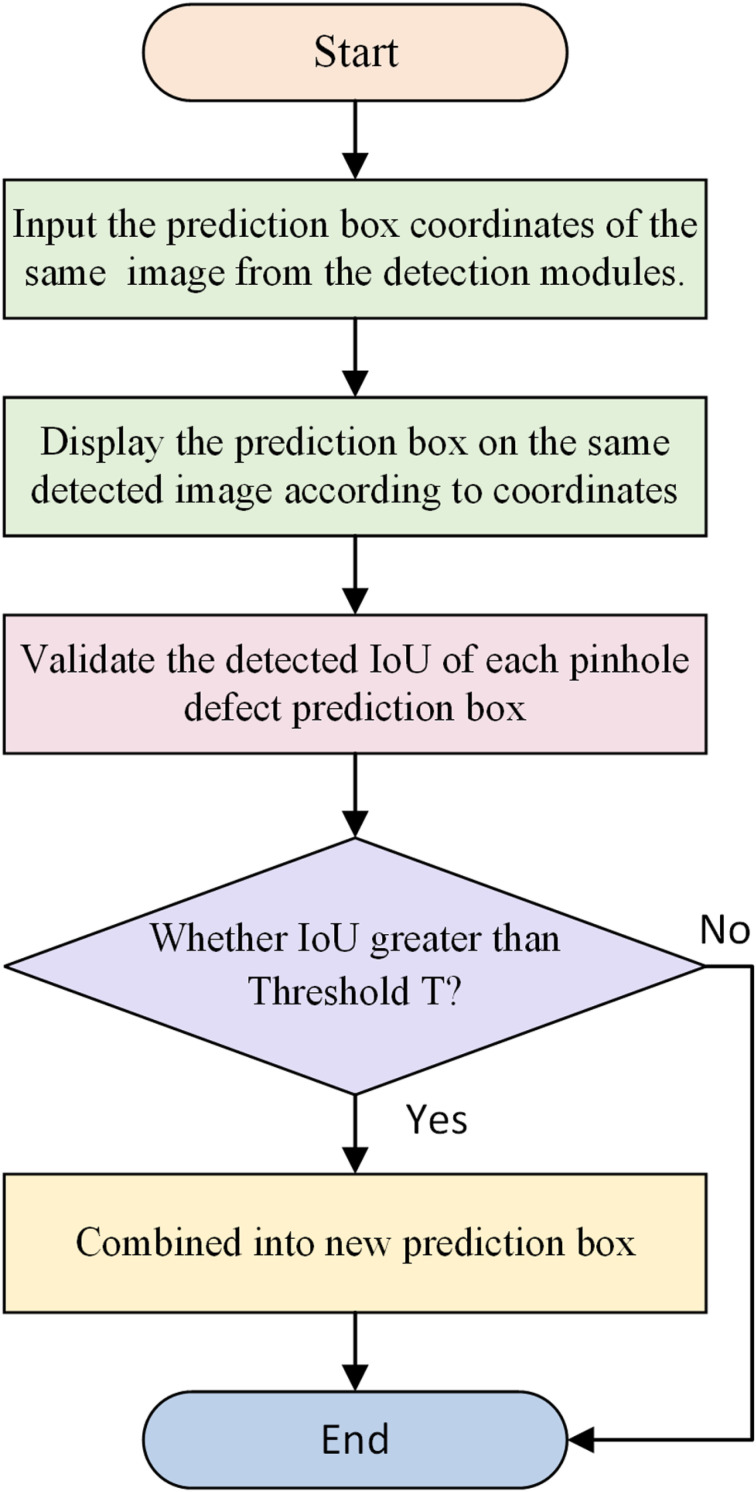
Verification flow of detection results of the two pinhole defect detection modules.

**Fig 11:**
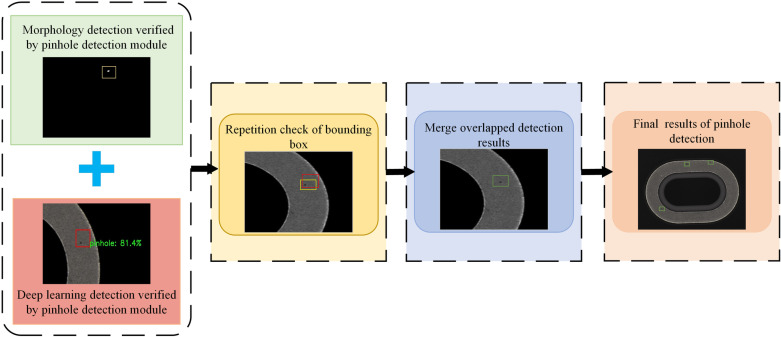
Fusion process of detection results of the two pinhole defect detection modules.

The function of the fusion module of pinhole defect detection result is to fuse the detection results after verification of the aforesaid two pinhole defect detection modules, so as to obtain the final and more accurate pinhole defect detection results. In the pinhole defect detection framework of this Paper, the inspection results of different modules may contain multiple responses to the same defect. Therefore, it is necessary to verify the detection results of the two detection modules, and the verification process is shown in (Fig 10). In this process, if the Intersection over Union (IoU) of the prediction bounding boxes obtained by different modules is larger than the IoU threshold X, the two prediction boxes are regarded as overlapping, and the overlapping prediction boxes are combined as responses to the same pinhole. Finally, the verified detection results of the two pinhole defect detection modules are fused, and the fusion process is shown in (Fig 11).

### 2.3 Implementation process of proposed method

**Fig 12:**
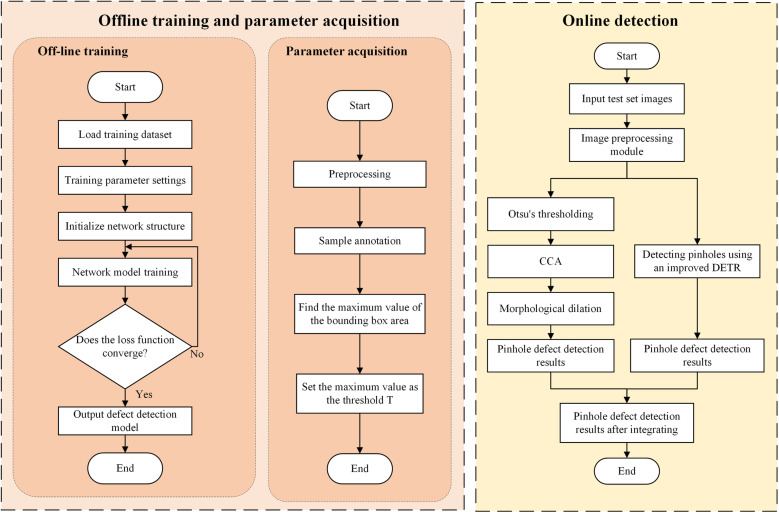
Implementation process chart of proposed method.

The implementation process of the method in this Paper is shown in (Fig 12), and it is divided into two stages: offline training & parameter acquisition and online detection. In the offline training, 1000 images are randomly extracted from 2000-frame original samples for the training stage, and the other original samples are used to evaluate performance of the method and do not participate in the training process. Then, data augmentation is carried out on aforesaid samples used for the training stage; after the number of samples used for training is enlarged to 3000 frames, the training set and the validation set are divided according to the ratio of 8:2. Since the number of training samples is still limited even after data augmentation, prior to training, the network is loaded with weights obtained by pre-training the public dataset ImageNet.

The parameter acquisition process aims at obtaining area threshold T of the connected component in the morphology-based pinhole defect detection module. The collected sample images are pre-processed to remove interference from noise; then, the preprocessed sample pictures are labeled, and the maximum value of the area of the labelled rectangular frame is found by algorithm and set as the area threshold T of the connected component in the morphology-based pinhole defect detection module.

The online detection stage is divided into two detection modules: morphology-based pinhole defect detection and deep learning-based pinhole defect detection. Firstly, the test set images are input into the preprocessing module, and then the preprocessed images are input into the two pinhole defect detection modules separately. The morphology-based pinhole defect detection module uses morphological methods to detect pinhole defects, and outputs the detection results of the morphology-based pinhole defect detection module after processing with Otsu’s method, CCA and morphological expansion. The pinhole defect detection module based on deep learning detects pinhole defects with improved DETR network. In addition to the information of the region where any defect may be located, the detection result output by the network also contains the confidence level of each defect region, and the low confidence threshold Z is set in this Paper, and the area with confidence lower than Z is not regarded as the area where the defect is located. Finally, the detection results of the two modules are verified, and the pinhole defect detection results after verification are fused.

## 3. Experiment and analysis

### 3.1 Experimental environment and evaluation indicators

The training parameters of the improved DETR network model are shown in Table 3. All the algorithms are carried out on the deep learning workstation using PyTorch 1.7.1 deep learning platform. The deep learning workstation uses Window10 operating system, the CPU is Intel(R) Xeon(R) Gold 6139M @ 2.30GHZ, the memory is 128G, and the GPU is NVIDIA GeForce RTX 3080.

**Table 3:** Training parameters of the improved DETR network model.

Training parameters	Pinhole defect detection network
Optimization algorithm	Adam
Pre-training weight	ImageNet
Initial learning rate	1e-5
Batch size	4
Training round Weight decay	100 1e-4

The other parameters of the proposed algorithm are set as follows: the area threshold T at the time of CCA is 150, the IoU threshold X in the verification mechanism is 0, and the confidence threshold Z of pinhole defect detection network is 0.01.

The reasonable selection of evaluation indicators is the key to measure the performance of the model. The focus of this study is to accurately detect defects, so the two indicators, namely, Precision (P) and Recall (R), are selected to validate the overall method, and the mean average precision (mAP) of each category is used to validate the impact of data augmentation on the performance of the object detection model [29,30].

Precision is the ratio of the number of correctly predicted positive samples to the number of samples predicted to be positive [31,32]. The formula is as follows:


$$P=\frac{TP}{TP+FP}$$ (3)

Recall is the ratio of correctly predicted positive samples to the number of positive samples [33,34]. The formula is as follows:


$$R=\frac{TP}{TP+FN}$$ (4)

Of which, TP represents the number of positive samples correctly classified, FP represents the number of negative samples misclassified, and FN represents the number of positive samples misclassified. According to the calculated Precision and Recall, the P-R curve is plotted, and the area enclosed under the curve is the Average Precision (*AP*). *mAP* reflects the global detection performance of the model, and is obtained by summing and averaging the average precision of a single class. The formula is as follows:


$$AP=\int_{0}^{1}P\left(R\right)dR$$ (5)

$$mAP=\frac{\sum_{i=1}^{N}R_{{AP}_{i}}}{N}$$ (6)

### 3.2 Effective validation of data augmentation

The defect detection method proposed by this Paper is a mixed model combining deep learning algorithms and traditional image processing algorithms, and the final detection result will be affected by several sectors; of which, traditional image processing method is independent of quantity of samples; using the overall methodology of this Paper directly, so it is difficult to clearly appraise the effectiveness of data augmentation. Therefore, this experiment uses original DETR network as the detection model of all types of defects of the backbone network to evaluate effectiveness of data augmentation.

The samples prior to data augmentation are original images used in the training phase mentioned in Subsection 2.3, while the samples obtained after data augmentation are 3000-frame pictures through data augmentation as described in Subsection 2.3. The images mentioned in Subsection 2.3 used for evaluating the method performance are used as a test set to verify the performance of the model before and after data augmentation, and Table 4 shows the mAP of the model before and after data augmentation. It can be observed from the table that after training the model with the data augmentation, the mAP of the model increases from 0.58 to 0.74, an increase of 16 percentage points, and the improvement of model precision indicates that the data augmentation method used in this Paper can effectively enhance the performance of the object detection model.

**Table 4:** mAP of the model before and after data augmentation.

Type of model	Use data augmentation	mAP/%
DETR	No	58
DETR	Yes	**74**

### 3.3 Fusion experiment of the improved object detection network

The purpose of the fusion experiment is to validate the effectiveness and advantages of the improved DETR network when it is applied to the detection of micro pinhole defects on the surface of metallized ceramic rings, and the dataset used in the training of each model is 3000-frame defect samples after data augmentation. In order to facilitate comparative analysis, four defect detection models which can detect pinhole defects in metallized ceramic rings at one time are established based on the DETR network architecture. Model 1 adopts the improved object detection network in this Paper. Model 2 restores the improved linear combination loss function in this Paper to the regression loss function of the standard DETR model prediction network. Model 3 not only restores the loss function, but also restores the improved attention module in this Paper to the attention module of the standard DETR network. Model 4, a standard DETR network, restores the improved backbone network to the ResNet50 used by the standard DETR network on the basis of Model 3. Settings of different models and comparison of detection accuracy are shown in Table 5.

**Table 5:** Settings of different models and comparison of detection results.

Model	EfficientNet-B2	SimAM	SmoothL1+CIoU	mAP/%
1	√	√	√	**79.0**
2	√	√	×	78.5
3	√	×	×	77.9
4	×	×	×	74.7

As can be seen from Table 5, the mAP of Model 1 is 0.5 percentage point higher than that of Model 2, indicating that the detection accuracy of the linear combined regression loss function using Smooth L1 and CIoU is higher. The mAP of Model 2 increases by 0.6 percentage point compared with Model 3, indicating that compared with the SE attention mechanism, the SimAM can better capture the subtle features of the defects because it estimates the 3-D attention weight and retains the effective information such as the spatial position of the defects in the metallized ceramic ring; although the aforesaid two improvements do not improve mAP greatly, the test sample size used in our evaluation is sufficient, and the result is reliable. The mAP value of Model 3 is increased by 3.2 percentage points compared with Model 4, which proves that the EfficientNet-B2 lightweight network can effectively avoid the problem of insufficient extraction of feature information of micro defects by ResNet50, and significantly improve the model’s ability to extract small targets. All the improvements to the standard DETR network in this Paper can improve detection performance of metallized ceramic ring defect to a certain extent. The improved object detection network in this Paper has the highest detection accuracy, reaching 79.0%, which is 4.3 percentage points higher than that of the standard DETR network. This is a significant improvement for the optimization of deep learning network.

### 3.4 Analysis of effectiveness of fusion of multiple modules

In order to validate the effectiveness of the complementary method of traditional image processing and deep learning designed in this Paper on improvement of the accuracy of pinhole defect detection, the following three pinhole defect detection methods are designed: Method 1 only uses the morphology-based pinhole defect detection module to detect pinhole defects; Method 2 only uses deep learning-based pinhole defect detection module to detect pinhole defects. Method 3 detects pinhole defects by fusion of aforesaid two detection modules. All methods use the original samples used to evaluate the performance of the methods mentioned in Subsection 2.3 as test images; Recall and Precision are selected as the evaluation indicators, and the detection results of different methods are shown in Table 6.

**Table 6:** Pinhole Detection Results of Different Methods.

Method	Recall/%	Precision/%
1	82.4	75.0
2	62.0	**88.9**
3	**83.5**	86.0

As can be seen from Table 6, Method 3 has the best detection effect, with the Recall of 83.5% and Precision of 86.0%. Method 1 performs well in Recall, but has a low Precision. Method 2 performs excellently in Precision, but its Recall rate is relatively low, with many missed detections. Method 3 has high Recall and Precision, indicating that the complementary method of traditional image processing and deep learning can more effectively detect the micro pinhole defects on the surface of metallized ceramic rings.

### 3.5 Evaluation through comparing with other methods

In order to further validate the effectiveness of the proposed method of this Paper in the pinhole defect detection task of metallized ceramic rings, this Section compares the proposed method of this Paper with the recent work in the detection of micro defects. Methods used for comparison include YOLOv8+RepLKNet DETR [35], Deformable-detr [36], YoloFD and BDD-DETR [37]. The test set pictures used to appraise performance of method, as mentioned in Subsection 1.3, are applied to compare and evaluate each method, and all models also use transfer learning to pre-train the network, Recall and Precision are selected as the evaluation indexes, and the detection performance and results of different methods are shown in Table 7.

**Table 7:** Comparison of performances of different detection methods.

Detection method	Recall/%	Precision/%
BDD-DETR	48.7	75.2
Deformable-detr	35.6	78.1
DETR	38.5	70.6
YOLOv8+RepLKNet	47.3	62.1
YoloFD	52.0	64.0
Proposed method	**83.5**	**86.0**

Table 7 shows the detection performance of the six detection methods on the defect test dataset. The method proposed in this Paper has the best performance of detecting pinhole defects on the surface of metallized ceramic rings, with Recall of 83.5% and Precision of 86.0%, both of which are optimal. The detection performance of the other five methods lags behind that of the proposed method in this Paper. Although these methods have relatively stable Precision, but have low recall, especially the Recall of Deformable-detr or DETR is less than 40%. In contrast, the proposed method in this Paper has obvious advantages in both Recall and Precision, indicating that it has the best comprehensive performance in detecting micro pinhole defects on the surface of metallized ceramic rings, and can better satisfy the detection requirements.

Table 8 shows the average inference time of different detection methods for a single-frame image. It can be seen from the table that although the inference time of the proposed method in this Paper for a single-frame image is a bit longer than that of the lightweight network YOLO, the proposed method can complete the defect detection of one frame image in about 0.14 seconds. The proposed method can achieve excellent detection effect without significantly increasing the inference time, and satisfy the real-time requirements of online detection.

**Table 8:** Single-frame image inference time of different detection methods.

Model	Average inference time/(Frame∙ms^-1^)
BDD-DETR	135.6
Deformable-detr	128.7
DETR	134.4
YOLOv8+RepLKNet	20.1
YoloFD	22.4
Proposed method	142.6

## 4. Conclusion

As an important and novel electronic part, metallized ceramic ring is widely used in communications, new energy, aerospace and other fields. The production process of metallized ceramic ring is complex, and defect detection is required to guarantee the quality of its finished product. Pinhole defect is a common surface defect in the metal layer of metallized ceramic rings. The pinhole defect is very small, and the background texture of the area where the defect is located is complex, so it is extremely difficult to detect pinhole defect. The prior art cannot reliably realize the automatic detection of pinhole defects of metallized ceramic rings, and actual production still mainly relies on manual detection, which is inefficient, labor intensive and effect uncertain. It is necessary to develop the technology of automatic detection of pinhole defects in metallized ceramic rings.

In view of the complex surface texture of the metal layer of metallized ceramic ring and tiny area of pinhole defect, this Paper sets up a pinhole defect detection framework combining traditional image processing with deep learning, and proposes a method for detecting small pinhole defects on the surface of metallized ceramic rings with improved DETR network and morphological algorithm. The main contributions of this Paper are as follows: 1. A micro defect detection framework integrating deep learning and morphology method is established to exert the advantages of both data-driven method and traditional image processing method. 2. Based on the characteristics of micro-object detection under background of complex texture, the DETR object detection network has been improved, and the accuracy has been significantly improved. Experimental results show that the accuracy of the proposed method in this Paper is significantly better than that of the existing mainstream small target detection methods, and the real-time performance meets the requirements of online detection. This study can also provide reference for the development of other small object detection methods.
